# Next Generation Sequencing Approach in a Prenatal Case of Cardio-Facio-Cutaneus Syndrome

**DOI:** 10.3390/ijms17060952

**Published:** 2016-06-16

**Authors:** Mafalda Mucciolo, Claudio Dello Russo, Laura D’Emidio, Alvaro Mesoraca, Claudio Giorlandino

**Affiliations:** 1 Department of Human Genetics, Altamedica Fetal-Maternal Medical Centre, 00198 Rome, Italy; mafalda.mucciolo@artemisia.it (M.M.); claudio.dellorusso@artemisia.it (C.D.R.); 2 Department of Prenatal Diagnosis, Altamedica Fetal-Maternal Medical Centre, 00198 Rome, Italy; laura.demidio@artemisia.it (L.D.); claudio.giorlandino@artemisia.it (C.G.)

**Keywords:** cardiofaciocutaneous, RASophaties, BRAF, next generation sequencing, NGPD

## Abstract

Cardiofaciocutaneous syndrome (CFCS) belongs to a group of developmental disorders due to defects in the Ras/Mitogen-Activated Protein Kinase (RAS/MAPK) signaling pathway named RASophaties. While postnatal presentation of these disorders is well known, the prenatal and neonatal characteristics are less recognized. Noonan syndrome, Costello syndrome, and CFCS diagnosis should be considered in pregnancies with a normal karyotype and in the case of ultrasound findings such as increased nuchal translucency, polyhydramnios, macrosomia and cardiac defect. Because all the RASopathies share similar clinical features, their molecular characterization is complex, time consuming and expensive. Here we report a case of CFCS prenatally diagnosed through Next Generation Prenatal Diagnosis (NGPD), a new targeted approach that allows us to concurrently investigate all the genes involved in the RASophaties.

## 1. Introduction

Cardiofaciocutaneous syndrome (CFCS) is a rare genetic disorder characterized by distinctive craniofacial features, congenital heart defects, failure to thrive, psychomotor delay and abnormalities of the skin and hair. Brittle, sparse and curly hair are key clinical features in CFCS [1]. Heart disease also is a postnatal common feature of cardiofaciocutaneous (CFC). The most frequent anomalies reported were pulmonary valve stenosis, hypertrophic cardiomyopathy, and atrial and ventricular septal defects [2].

CFCS shares overlapping clinical features with other conditions collectively named RASopathies. RASopathies include Noonan syndrome (NS), Costello syndrome (CS), and cardiofaciocutaneous syndrome (CFCS), as well as LEOPARD syndrome (LS), neurofibromatosis type 1, Noonan syndrome with loose anagen hair (NS/LAH), Legius syndrome and neurofibromatosis-Noonan syndrome. Collectively, RASopathies have a prevalence of between 1 in 700 and 1 in 1250 live births [3] and are caused by the deregulation of the Ras/Mitogen-Activated Protein Kinase (RAS/MAPK) signaling pathway. This pathway plays a key role in the control of the cell cycle, but also in differentiation, growth and cell senescence during embryonic and postnatal development. The germ line mutations in gene encoding proteins of the RAS/MAPK pathway have a gain of functional character and cause the constant activation of the pathway. This leads to disturbances in the development of the neural crest derived cells, improper cell migration or increased cell proliferation.

NS is associated with mutations in *PTPN11*, *SOS1*, *KRAS*, *NRAS*, *RAF1*, *BRAF*, *SHOC2*, *MEK1* and *CBL* [4,5,6,7,8,9,10,11], CS with mutations in *HRAS* [12], CFCS with mutations in *KRAS*, *BRAF*, *MEK1* and *MEK2* [13,14], NS/LAH with mutations in *SHOC2* [10], LS with mutations in *PTPN11*, *RAF1* and *BRAF* [4,8,9].

Recently, new additional genes, including *RIT1*, *RRAS*, *RASA2*, *SOS2*, *A2ML1* and *LZTR1*, have been shown to be associated with RASophaties [15]. The *RIT1* gene is involved in modulating neurite outgrowth and activating the extracellular signal-regulated kinase (ERK) and p38 MAPK [16,17]. *RRAS*, *RASA2* and *SOS2* are functionally associated to the RAS/ERK pathway [15].

Due to the genetic heterogeneity and the high variability in clinical signs, establishing a diagnosis of these disorders is often difficult. Patients are generally diagnosed postnatally, but prenatal characteristics are also described in literature. The prenatal manifestations of NS were described by Benacerraf [18] and Nisbet [19] more than 20 years ago. They include increased nuchal translucency (NT), cystic hygroma, polyhydramnios, cardiac defects and pleural effusions [20]. In CS, the more documented prenatal signs are polyhydramnios, cardiac anomalies and arrhythmia, and relative macrocephaly [21]. Little information is reported that describes the perinatal course of CFCS. Myers and colleagues recently reported a cohort of nine fetuses with postnatal diagnosis of CFCS [22]. The most common prenatal features were polyhydramnios (89% of cases), renal anomaly (55%), lymphatic dysplasia (22%), fetal abdominal circumference >90° centile (22%) and congenital heart defect (11%).

Here we report a case of CFCS prenatally diagnosed by genetic amniocentesis in a fetus with first trimester cystic hygroma.

## 2. Results

### 2.1. Case Report

A primigravida woman, 19 years old, was referred at 17 weeks of gestation to our Fetal Medical center because the presence of nucal cystic hygroma diagnosed during an office ultrasound scan performed at 13 weeks of gestation. Maternal and familiar medical history were unremarkable, non invasive prenatal testing (NIPT) that was performed at 11 weeks of gestation resulted in low risk fetus for trisomy 21, 13, 18.

Our ultrasound examination confirmed the presence of cystic hygroma but no other abnormalities were noted; the biometry was regular for gestational age. Follow-up sonography at 21 weeks of gestation revealed persistent nucal cystic hygroma, polyamnios (maximum vertical pockets of amniotic fluid 92 mm), cefalic and abdominal biometry above the 95° centile for gestational age, femur length at 10° centile, bilateral borderline ventriculomegaly (10,5 mm), bilateral hyperecoic kidneys. No abnormalities were seen at the prenatal echocardiography. Clinical signs shown in the present case completely overlap those prenatally reported in CFCS cases in the literature (Table 1).

According to the couple’s wishes, we decided to perform a genetic amniocentesis and to schedule the subsequent steps based on the results of genetic analysis.

### 2.2. Next Generation Sequencing Analysis

**Figure 1:**
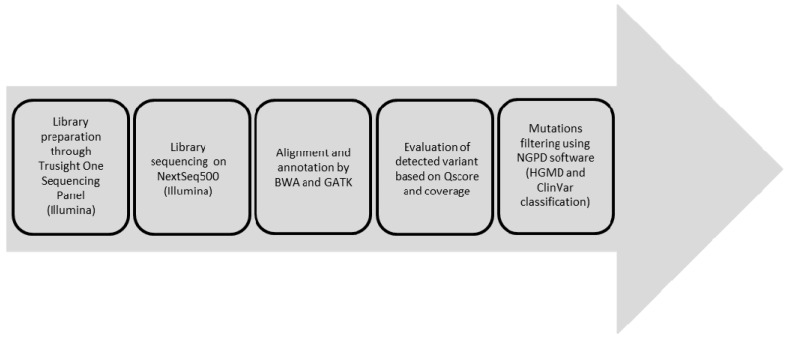
Experimental workflow.

The presence of chromosomal anomalies was ruled out by standard karyotype (46, XY). We then proceeded with NGPD analysis on DNA extracted from amniotic fluid sample. Sequencing analysis was performed using the TruSight One Sequencing Panel (Illumina, San Diego, CA, USA) and next-generation sequencing on NextSeq (Illumina). After sequencing, we investigated only 300 genes whose mutations are responsible for approximately 100 well-known pathologies [23] (Figure 1), including the genes associated with RASophaties.

**Figure 2:**
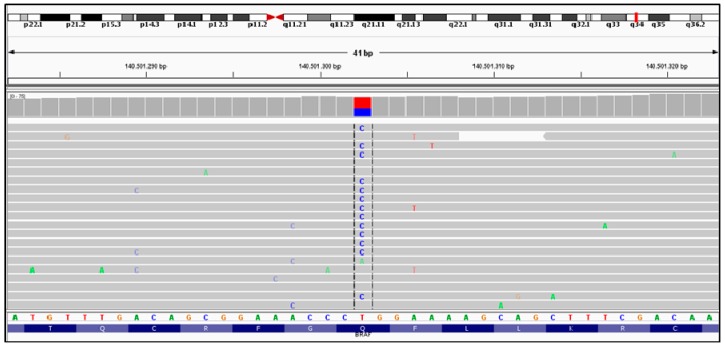
*BRAF* mutation (p.Q257R) visualized via Integrative Genome Viewer (IGV). Dashed lines show nucleotide substitution; red square represents the percentage of wild nucleotide (T); blue square represents the percentage of alternative nucleotide (C).

NGPD analysis revealed a mutation in exon 6 of *BRAF*, denoted p.Q257R (c.770A>G) (Figure 2). This mutation was previously associated with CFCS [13,24]. Given the autosomal dominant form of the disorder, the fetus was confirmed to be affected by Cardiofaciocutaneous syndrome. No additional pathogenetic mutations were found through the analysis of the sample.

Because the clinical suspicions was also confirmed by molecular analysis, a multidisciplinary counseling (genetic, obstetric and pediatric) was offered to the couple, who decided to terminate the pregnancy.

## 3. Discussion

The term RASopathy refers to a group of heterogeneous genetic disorders that share similar phenotypes and are caused by mutations of different genes of the RAS/MAPK pathway. The shared features make the molecular characterization of RASophaties complex, time consuming and expensive. Diagnosis of RASopathies is usually made postnatally and many articles reported perinatal features in these cases. NS is a more frequent RASopathies; therefore, its prenatal features have been better defined. Increased nuchal translucency or cystic hygroma (30%–53%) and polyhydramnios (38%–57%) are commonly reported in NS [25]. In CS, marked polyhydramnios, fetal overgrowth and relative macrocephaly are diagnostic criterions [21]. Less data are available on prenatal characteristics of CFCS. However, the pattern of developmental anomalies *in utero* is similar among the three conditions. Polyhydramnios is reported in approximately 50% of CFC patients and seems to be responsible for premature birth [2]. Additional prenatal ultrasound signs reported in CFC patients are hydronephrosis, cerebral ventriculomegaly, macrosomia, cystic hygroma, and relatively short femora [2,24,26,27]. In CFCS, as well as NS and CS, cardiac defect, reported postnatally in more than half of patients, are undiagnosed *in utero* [28]. The typical cardiac malformations of CFCS (as pulmonary valve stenosis, hypertrophic cardiomyopathy, atrial and ventricular septal defects) are late onset and progressive and therefore their prenatal detection is difficult. The fetus presented in our case report showed the same prenatal sonographic signs as reported in the literature and related to CFCS. Also, in our case, cardiac malformations were not detectable at 21 weeks.

Through NGPD analysis the pathogenic mutation Q257R was identified in *BRAF*. This mutation is the most common and represents about 25% of all *BRAF* mutations. Germline mutation in *BRAF* have been identified in about 75% of patients with clinical suspicion of CFCS. *MEK1* and *MEK2* mutations account for the 20%–25% of cases and *KRAS* for less than 5%. About 80% *BRAF* mutations are missense and confer an activation of the oncoprotein. *BRAF* mutationsin exon 6 (p.Q257R), in exon 12 (p.E501*), and in exon 11 (p.G469E) are the most commonly reported [29]. Long-term data available for patients with Q257R mutation showed severe intellectual disability, seizures, pulmonic stenosis and abnormal brain MRI [24].

## 4. Materials and Methods

### 4.1. Library Preparation

For the library preparation, we used an enrichment method developed by Illumina which is composed of 4813 genes, with a cumulative target region size of 12 Mb, and the probe set was designed to enrich for about 62,000 exons (TruSight One Sequencing Panel, San Diego, CA, USA). The genes included in the library were selected by Illumina based on the Human Genome Mutation Database (HGMD), the Online Mendelian Inheritance in MAN (OMIM) catalog, GeneTests, Illumina Trusight panels, and other sequencing panels. The use of such a kit prevents the development of a library for each single gene of interest using, instead, the evaluation of the producer who established a target minimum coverage value of 20×. The Trusight one sequencing panel kit contains all the reagents necessary for library preparation and also for the use of the NextSeq 500 sequencer (Illumina). Experimental workflow was realized according to the manufacturer’s instructions. Library sequencing was performed on NextSeq500 platform (Illumina).

### 4.2. Data Analysis

Data analysis was performed as previously described by Dello Russo *et al*. [30]. Briefly, NextSeq500 platform provides a primary data analysis. Basespace on site software (Illumina) performed secondary analysis on the base calls. Basespace also produced a Phred-like quality score (Q score), optimized run conditions and provided run-time quality statistics. The Trusight one sequencing panel workflow required a reference genome that provided variant annotations and set the chromosome sizes. Single nucleotide polymorphisms (SNPs) and short indels were identified by Genome Analysis Toolkit software (GATK, Broad Institute, Cambridge, UK). The identified variants were evaluated for coverage and Q score. All regions reported with a sequencing depth <30 were classified as unsuitable for analysis. Variant Studio software (Illumina) was used for variant calling and HGMD professional and ClinVar NCBI database were used for variants classification.

## 5. Conclusions

In conclusion, the prenatal period of CFCS presents no peculiar ultrasound findings with respect to Noonan and Costello syndrome. Therefore, if chromosomal abnormalities are not reported, NS should be considered as possible diagnosis in pregnancies with prenatal ultrasound findings of polyhydramnios, increased NT, cystic hygroma, persistent nuchal fold, pleural effusion and cardiac defects. The presence of fetal macrosomia and increased head circumference could otherwise support the diagnostic suspicious of all the three disorders, NS, CS, or CFCS.

However, an ultrasound based diagnosis of CFCS is difficult because of the shared signs with other RASopathies, particularly prenatally and in newborns. Sanger sequencing is currently the gold standard method for the molecular characterization of patients suspected of RASophaties. However, due to the huge number of genes and the absence of mutational hot spots, defining “gold strategies” for the management of these patients is difficult. While molecular diagnosis of CS is ruled out in absence of HRAS mutations, due to genetic heterogeneity, molecular diagnosis of CFCS is long and time consuming. Moreover, the technical advances of the last years, led to the identification of new genes that could be responsible for RASophaties and that therefore need to be screened when the clinical suspicious of NS, CS or CFCS is present. The discovery of these new genes involved in the RASophaties suggest the need to continuously update the sequencing kits in order to better define the frequency and the recurrence risk of the diseases and possibly define new genotype–phenotype correlations.

The strategy we reported here showed, however, a new sequencing approach for prenatal diagnosis that allows us to concurrently investigate the 11 genes principally involved in the RASophaties until now, consistently with timing provided by prenatal diagnosis but also with low quantity and quality of DNA extracted from the prenatal sample.

## Figures and Tables

**Table 1:** Prenatal signs in Cardiofaciocutaneous Syndrome.

Cases	HC	AC	FL	P	LD	CHD	RA
Present Case	present	present	present	present	present	not present	present
Allanson 2011	n.a.	22/64	n.a.	85/132	n.a.	n.a.	n.a.
Myers 2014	1/9	2/9	n.a.	8/9	2/9	1/9	5/9
Templin 2015	8/11	9/11	5/13	10/15	2/12	0/15	7/15
Total	48%	40%	43%	66%	23%	4%	52%

HC = Head Circumference > 90°–95° centile; AC = Abdominal Circumference > 90°–95° centile; FL = Femur length < 10° centile; P = Polyhydramnios; LD = Lymphatic dysplasia; CHD = Congenital Heart Disease; RA = Renal Anomalies; n.a. = not available.
